# Biobank consent preferences and implications for a mixed consent model: biomedical researchers vs. community stakeholders in a semi-urban Yoruba community, Nigeria

**DOI:** 10.3389/fgene.2026.1779086

**Published:** 2026-06-03

**Authors:** Olubunmi A. Ogunrin

**Affiliations:** Biomedical Ethics Research Group, Department of Health Services Research, University of Liverpool, Liverpool, United Kingdom

**Keywords:** biobanking, communitarianism, consent preference, ethical preference, informed consent, Nigeria

## Abstract

**Background:**

Several consent models have been described in the literature for genomic research, with some focusing specifically on biobanking. Sub-Saharan African scholars reported a preference for broad consent among key stakeholders, identical to narratives from most studies in Europe and the USA. However, there have been reports of a generational shift with divergent views among potential genomic research participants in sub-Saharan Africa due to communitarian ethos and relative solidarity. To avoid ethical conflicts in biobanking research in sub-Saharan Africa, it is imperative to explore the preferences of the various stakeholders.

**Objective:**

To explore the opinions of research stakeholders, namely: biomedical clinician-researchers, community elders, and community members, on informed consent models in biobanking research.

**Methods:**

This qualitative study employed key-informant semi-structured interviews and focus group discussions to collect data from purposively selected participants. Sample sizes for the stakeholders’ categories were determined by theoretical saturation. Thirty clinician-researchers and four community elders were interviewed. Fifteen focus group sessions were held with 50 community members. The methodological design, adapted from grounded theory, used the constant comparative method of data analysis. Data and methodological triangulation, reflexivity, and code-recode reliability index were used to ascertain data quality.

**Results:**

Twelve of the biomedical researchers preferred blanket consent, aligning with the preferences of adult community members. Ten of the researchers opted for broad consent. The community elders opined that community members would prefer tiered consent. The youth participants differed from the researchers and community adults, preferring re-consenting. The findings of this study showed discordant views on consent model preferences among the various stakeholders.

**Conclusion:**

Discordance in consent preferences among the key stakeholders is a potential source of ethical conflict. A hybrid or mixed-consent model that provides participants with the option to choose the consent model they prefer for every research stage, and flexibility to change their choices as the research progresses, is recommended. This will reflect the fundamental principle of autonomy and demonstrate responsive communitarianism and relative solidarity. It will also provide a robust, culturally sensitive, and context-specific model that reflects the preferences of community stakeholders and addresses the fundamental ethical issues encountered in biobanking.

## Introduction

Informed consent is defined as ‘receiving information necessary to make an informed choice about research participation, understanding that information, and making a voluntary decision on whether to participate’ (Council for International Organizations of Medical Sciences CIOMS in collaboration with World Health Organization, 2002). Effective and acceptable informed consent is a *sine qua non* for ethical clinical trials and health services research, and the key mechanism for protection of human participants in research from harm and exploitation. Furthermore, it serves as the ‘moral contract’ between the researcher and study participants and should be seen as a process rather than the research participant simply signing the consent form (Khan et al., 2014).

Consent is a process that commences from the recruitment of research participants through to the completion of research, as it may become necessary to seek re-consenting or utilise a tiered or stepwise consent method while the research is ongoing. Research Ethics Committees (RECs) require that effective and voluntary informed consent be obtained before a prospective participant is enrolled in research (Anderson et al., 2012; Bean et al., 2010). The emphasis on a person’s right to accept or refuse to participate in biomedical research reflects important ethical principles such as respect for human dignity and autonomy. It is anchored on the principle of the fundamental individual right to autonomous decision, and unrestricted access to pertinent and correct information that aids in decision-making (Miller et al., 2011).

The ingredients required for decision-making include rationality, freedom from external forces (that is, an individual’s autonomy and freedom from coercion), adequate understanding of the subject matter (that is, comprehension) and the individual’s competence. The competence of the individual refers to the ability of a person to perform a task, the ability to understand the type, duration, risks, benefits and procedure of research (or in a clinical setting - diagnosis, prognosis, risks and benefits of treatment or procedure), the ability to act or do the same thing consistently and the ability to communicate concerns and choices (Afolabi et al., 2014; Zabow, 2008). However, the process of consent is complex as it is influenced by many factors such as culture, religion, societal norms, literacy, and personal identity. For example, the cultural norms and practices may influence community members’ decisions to participate in research. Mystakidou et al. (2009) in a systematic review, demonstrated that cultural barriers due to communitarianism, illiteracy, language barriers or lack of true understanding of the entire study, and diminished autonomy were some of the ethical and practical challenges in implementing informed consent in HIV/AIDS clinical trials in resource-limited countries (Mystakidou et al., 2009).

Furthermore, some authors have observed the disparity in the consent process in international collaborative research involving developed and developing countries, whereby the informed consent process used is incompatible with the socio-cultural setting of the study participants recruited from the indigenous communities (Dawson and Kass, 2005; Izadi et al., 2012). In the setting of biobanking research, adhering to cultural beliefs and practices regarding donating any body part, including blood, in sub-Saharan Africa poses challenges to informed consent and research participation, and may require different approaches (H3Africa Consortium et al., 1979; Wright et al., 2013). This becomes more obvious in resource-poor settings where a lack of ethical regulatory bodies can result in poor capacity for ethical review and oversight of research. When research sites are situated in these resource-limited countries, ethical guidelines stipulate that independent ethical review by a local ethics committee is mandatory (Nuffield Council on Bio ethics, 2002; UK Research Integrity Office, 2009; World Medical Association, 2008). The lack of ethical regulation of research in developing countries paves the way for the adoption and application of ethical guidelines from developed countries, and such guidelines may reflect moral values and standards different from those of the developing countries, which, when applied to research in the developing countries, inadvertently result in unethical and poorly conducted research.

Several consenting models regarding biobanking have been described in the literature, namely blanket consent, broad consent, re-consenting, tiered consent, third-party oversight or delegate model, presumed consent, and dynamic consent (Appelbaum, 2017).

*Models of Consenting:* Many people view broad and blanket consent to be similar and use terms interchangeably, but they are not the same (McGuire and Beskow, 2010; Beskow et al., 2010; Beskow et al., 2014). Blanket consent refers to a process by which individuals donate their samples *without any restrictions,* while broad (also called “general”) consent refers to a process by which individuals donate their samples for a broad range of future studies, *subject to specified restrictions* (Wendler, 2013). An example of broad consent is when biobank donors insist on ethics committee approval as a specified restriction before sample re-use, especially if there are perceived risks, even when they have actively consented once for the current study and all future research involving the general use of their samples and information (other terminologies include open and generic). Contrariwise, in blanket consent, no ethics committee approval is sought for re-use, as there is no specified restriction (Haga and Beskow, 2008; van Schalkwyk et al., 2012/07; Tomlinson, 2013; De Vries et al., 2016).

Re-consent demands that biobank donors are informed and required to consent to the current study and then subsequently consent to each future research study involving the use of their samples and information (Ludman et al., 2010; Mee et al., 2013; Steinsbekk and Solberg, 2011). Hence, the researcher returns to obtain consent from research participants after obtaining the initial consent at the beginning of the research. Consent is presumed when donors allow the use of their samples and information for all research unless they actively opt out (Yassin et al., 2010; Auray-Blais and Patenaude, 2006; Kaye et al., 2014; D’Abramo, 1978). Third-party oversight implies that donors can actively consent, but an ethics board must approve before the commencement of every research using their stored samples and information. This third-party oversight is emerging as a common component of biobanking governance schemes (Yassin et al., 2010; Auray-Blais and Patenaude, 2006).

Dynamic consent involves giving a role to communities’ consultation, as part of the decision-making process, a model termed ‘active citizenship’. This is a patient or donor-centred approach that uses the mechanism of governance through information technology (IT) solutions and allows participants to engage as much as they choose and to alter their consent choices over time (Kaye et al., 2014). This approach can improve public trust, limit participants’ withdrawal from research, overcome the perennial issue of consent form length and comprehension, improve transparency and accountability in the research process through continuous contact with patients. This allows researchers to gather phenotypic information, reduce research biases, and, by using an epigenetic model, reduce the burden of social and environmental effects on health. This improves research participation, although it entails higher management costs (D’Abramo, 1978; D’Abramo et al., 2015a; Steinsbekk et al., 2013a).

Controversies exist as to the appropriate consenting process for biobanking, and it remains unclear if a single consent model should be adopted for biobanking research. For instance, the World Health Organisation and United Kingdom Human Genetics Commission proposed that public participation in genomic or biobanking research should assume that participants should be content with blanket consenting due to financial and logistical concerns (Murphy et al., 2009). On the other hand, the H3Africa Working Group recommended broad consent for the conduct of genomic research in sub-Saharan Africa (H3 Africa Working Group, 2011). These two examples demonstrate conflicting recommendations for the conduct of genomic or biobanking research. This underscores the need to explore the preferences of the stakeholders in biobanking research to avoid ethical dilemmas, especially in developing countries, where there already exist potential challenges, such as cultural and superstitious beliefs associated with donating body parts or fluids for this type of research (Peeters Grietens et al., 2014).

Several studies conducted among key biobank research stakeholders in sub-Saharan Africa reported a preference for broad consent (Maseme et al., 2024; Tindana and de Vries, 2016). It is unclear whether this recommendation reflects the views of biobank donors (patients/public) or is solely based on the African communitarian ethos. Communitarianism, according to Callahan (2003), is an ethical concept that assumes that human beings are social animals, not isolated individuals, whose lives are lived out within deeply penetrating social, political and cultural institutions and practices. This communitarian disposition among sub-Saharan Africans suggests that communal values are likely to affect the consent process and research participation. It stresses the significance of social bonds and the balance between individual rights and social responsibilities (Callahan, 2003). Ogunbanjo and van Bogaert (2005) identified two forms of communitarianism in Africa, radical (or authoritarian) and moderate (or responsive) communitarianism (Ogunbanjo and Knapp van Bogaert, 2005a; Ogunbanjo and Knapp van Bogaert, 2005b). Authoritarian communitarianism emphasises communal wellbeing and harmony over individual rights and autonomy, thus prioritising communal needs over individual interests to enforce social order. On the other hand, responsive communitarianism encourages a balance between communal responsibilities and individual rights, thus seeing the society not as an aggregate but as a community of individuals (Ogunbanjo and Knapp van Bogaert, 2005a; Etzioni, 1996).

Broad consent is the model permitted for biobanking research in South Africa (Maseme et al., 2024). It was suggested that a specific consent model would be challenging to put into practice (Mikkelsen et al., 2019). Tindana and de Vries (2016) advocated for a broad consent model for genomic and biobanking research in sub-Saharan Africa because it allows for the future use of biospecimens and associated data that may be unrelated to the original research. They argued that broad consent enhances a robust governance framework for biobanking and promotes global health and research equity (Tindana and de Vries, 2016). This ethical stance may be acceptable to the biobank researchers and managers. It remains questionable if it is acceptable to the community stakeholders within the ambit of responsive communitarianism and relative solidarity.

Ethical dilemmas may arise from differing preferences regarding consent models. I argue that to forestall conflicts in the conduct of genomic or biobank research, it is crucial to address the opinions of all stakeholders regarding informed consent preferences, especially prospective research participants, rather than recommending a particular model without considering their opinions. This becomes an important issue because of the generational shift and ethical concept of relative solidarity demonstrated by the youth participants in a Nigerian study; they chose to participate in research based on their personal convictions without neglecting the communal values and benefits (Ogunrin et al., 2018a).

Relative solidarity is an ethical concept of communal participation described in a sub-Saharan African context where there is alignment with the common good based on personal conviction rather than strict conformity to traditional communal norms (as seen in authoritarian or radical communitarianism) (Ogunrin et al., 2018a). This concept recognises divergent views within a community and allows balancing of shared interests with individual agency. Therefore, the recommendation and implementation of a particular consent model represent an authoritarian stance. This is incompatible with a flexible implementation of a consent model that allows for stakeholders to exhibit individual liberalism or relative solidarity.

No doubt, there is a dearth of literature on informed consent preferences among biobanking research stakeholders in Africa, especially with the growing impact of the H3Africa project on the continent. Globally, the literature on consent preferences in biobanking and genomic research is predominantly studies from Europe and America. Most of the research surveyed public perspectives on consent for biobanking, while very few studies explored the views and opinions of scientists (Master et al., 2014). This qualitative study, therefore, sought to explore the opinions and beliefs of the research stakeholders, namely, community members, community leaders, and biomedical researchers (clinician-scientists) from a defined indigenous African community, as regards the type of consent model they prefer when deciding to participate in biobanking research. The consent covers the use and storage of their biospecimens (body fluids–blood and saliva, body parts–organs) for initial and future research, including use and storage of their associated data.

## Methods

The data reported here is part of a larger qualitative study into informed consent and community engagement in genomic research. The details of the larger study’s objectives have been discussed in a previous publication (Ogunrin et al., 2018b). This paper focuses on the type of consent models preferred by the various research stakeholders. To achieve this goal, we interviewed biomedical researchers, community rulers and opinion leaders, and conducted focus group discussions with the community members.

### Ethical considerations

The Research Ethics Committee in the developing country of study (Reference number ADM/DCST/HREC/1792) and the Institutional Research Ethics Committee of a United Kingdom institution (Reference number IPHS-1415-LB-270) approved the research protocol. Written informed consent was obtained from all study participants, and verbal informed consent (with evidence of thumb printing) from participants who were not literate. Consent was also obtained for audio recording of the interviews and focus group discussions. Data obtained were de-identified to ensure participants’ privacy and confidentiality. Pseudonyms were used to represent participants for reasons of confidentiality.

### Study design

As this study explored the views and opinions of purposively selected participants on their preferences for informed consent models perceived to be appropriate for biobanking research, the methodological design was adapted from grounded theory (Glaser et al., 1967), and used the constant comparative method of data analysis (Silverman, 2000; Silverman, 2001). Focus group discussions (FGDs) were conducted with four categories of participants, and data were collected using an iterative process for each FGD. This process continued until theoretical saturation (Carlsen and Glenton, 2011; Curry et al., 2009; Mack et al., 2011) was reached for each category of participants, when no new or relevant data emerges.

### Study area

This research was situated at a tertiary health research institution in a semi-urban community in southwest Nigeria. It is a community of mostly Yoruba-speaking people with Christian, Islamic or Traditional religious affiliation. Employment is both white-collar (salaried) and indigenous traditional occupations like hunting, farming, and crafts.

### Study participants

Participants were purposively selected from community members attending the research facility. All study participants resided within the community and gave voluntary, informed consent to participate in the study. Biomedical researchers engaged in human subjects’ research at a research institution situated in the selected community participated in key informant semi-structured interviews. Community leaders participated in key informant interviews (face-to-face, semi-structured interviews).

The criteria for participation in the study included a) Membership of the community where the study was conducted, b) Provision of written or verbal (with thumb printing) informed consent, c) Ability to understand English and/or Yoruba language, and d) Availability to participate in FGD and/or interview session. The exclusion criteria were: a) Non-membership of the communities where the study was conducted, b) Failure to give informed consent, c) Non-availability to participate in FGD and interview sessions, d) Individuals less than 18 years of age, and e) Presence of mental or speech disabilities (which were determined by the study investigator through mental state examination of the potential study participants). Sequential or recursive sampling, which occurred throughout the life of the research, until theoretical saturation was achieved, was employed. The selection of study participants was not based on the inclusion and exclusion criteria, but rather to ensure that individuals not from the study site were excluded, since such individuals might have socio-cultural characteristics distinct from those of the potential study participants.

### Focus group methodology

The FGD participants were informed of the research details and gave consent. Those who were literate and could read were given an information sheet and the opportunity to ask questions about the research. Those who could not read were given the research details verbally by the interviewer (OO). As much as possible, recruitment occurred at first contact, as it is more difficult to follow up and get the potential participant to consent subsequently. Details of this process have been reported in a previous publication (Ogunrin et al., 2018b). Focus group discussions were conducted with either four or six participants at a time. Separate groups for the males and females were chosen for the FGDs to prevent gender-related authority influence, thereby minimising paternalism, which may affect women from freely expressing themselves in a mixed group, as noted previously by Fagbemiro *et al* (Fagbemiro and Adebamowo, 2014). The participants were encouraged to deliberate on their consent preferences during the focus group discussion sessions.

### Interview process

The biomedical researchers and community leaders were interviewed at pre-arranged, convenient times after obtaining their consents. On the day of the interview, the interviewer’s introduction (without details of professional status or academic background) and the purpose of the interview preceded the main interview. It was emphasised that there were no right or wrong answers; the goal was to obtain their honest views, opinions, and choices on the mode of consent they agree with when participating in biobanking research. Information on the various consent types was not provided in advance of the interview, so that they were at liberty to describe their preferences. The interview topic guide, though used, was not strictly adhered to; this allowed the interview to flow as each interviewee answered each question. The interviewees were encouraged to share their experiences from previous research and other relevant life experiences.

### Data analysis

The interviews were tape-recorded and transcribed verbatim daily into text, and transcripts were imported into Atlas. ti for initial coding of the data using a coding frame. During the data interaction and initial coding process, areas that required further clarification and probing were identified, and these guided subsequent interviews. Data were iteratively analysed as themes evolved during the analytic process; these were further probed at subsequent interviews and FGD sessions to achieve saturation and clarity. The initial or open coding yielded themes which were subjected to selective coding to identify common and explanatory categories for common themes. Deviant cases were identified, discordant views were deliberated on, and the quality of data was ascertained by data (informant) triangulation, methodological triangulation, code-recode analysis and reflexivity. The details of code-recode analysis (reliability index of 0.87) were presented in a previous publication (Ogunrin et al., 2018b).

## Results

### Study participants

Thirty biomedical researchers, comprising 16 males and 14 females, with a mean age of 40.4 (SD 5.4) years and an age range of 33 to 56 years, were interviewed. Most of them were involved in clinical science research; only three engaged in laboratory-based genetic studies. Details of the demographic data of the biomedical researchers are presented in Table 1. All four community leaders present at the study site were interviewed. All the interviewees were of Yoruba ethnicity and resided within the community. Fifteen focus group sessions were conducted with 50 community members, segmented by age and sex into four categories, namely, adult males, adult females, male youths, and female youths. Adult participants were above 30 years of age and married, while the youths were between 18 and 30 years of age. Nine of the 23 youths were married. The summary of study participants is presented in Table 2.

**TABLE 1:** Summary of demographics of biomedical researchers.

Variables	Distribution	Frequency	Comments
Age distribution	*Male* 31–40 years 41–50 years 51–60 years *Female* 31–40 years 41–50 years 51–60 years *Total* 31–40 years 41–50 years 51–60 years	8 6 2 11 3 0 19 9 2	Male Mean: 42.4 (SD 6.2) Median: 40.5 Range: 35–56 years Female Mean: 37.5 (SD 3.1) Median: 38 Range: 33–43 Total Mean: 40.4 (SD 5.4) Median: 39.5 Range: 33–56 years
Sex distribution	Male Female	16 14	​
Level of education	Primary Secondary Tertiary Postgraduate	- - - 30	​
Area of research	Basic sciences Clinical sciences Genomic research	8 19 3 (laboratory-based)	​
Ethnicity	Yoruba	30	​

**TABLE 2:** Summary of all study participants.

S/N	Participants’ category	Sex distribution		Total
		Female	Male	
1	Prospective research participants (FGD)	24	26	50
2	Community leaders	1	3	4
3	Biomedical researchers	14	16	30
​	Total	39	45	84

Community members and biomedical researchers agreed that informed consent must be obtained before participation in biobanking research, but they differed on the acceptable consent model. Based on their responses, the major themes were labelled using the existing consent models that best described their preferences, and these included: a) blanket; b) broad; c) re-consent; d) presumed; e) multi-layered or tiered; f) delegated trustee or third-party oversight; and g) dynamic models.

### Opinions of the biomedical researchers

The biomedical researchers differ in their preferences for an effective, appropriate model of consenting for biobanking research. Twelve of the biomedical researchers (40%) agreed that blanket consent is acceptable for biobanking research. This type of consent ignores the fact that samples may be reused for studies that conflict with an individual’s fundamental values (Tomlinson, 2013). One of the biomedical researchers put it this way, *‘Many Nigerians give their samples and are not bothered. Once Nigerians give, they give’* ***(Dr Sugar).*** One of the researchers with experience in community genetics research opined that ‘once they gave their blood samples, then they gave without attaching conditions to it’ and would not ask about the outcome of the research; *‘Once they have given their samples, they do not care what you do with it, but I think the onus lies on the researcher to stick to the terms of reference. But most people, once they give the sample, they forget it and continue with their life; in fact, they do not even care if you come back to tell them the results of your findings’ (* ***Dr Shaw).***

Thirty per cent (9/30) of the researchers expressed a preference for broad consent. The researchers who preferred broad consent specified ‘best interest of the participants’ as a restriction, that is, reuse of biospecimens accompanied by risks is not acceptable. One of these biomedical researchers used the surgical consent obtained pre-operatively in the clinical setting to illustrate this, in which the patient consents to ‘leave the surgery to the discretion of the surgeon in case of any eventualities, provided it is in best interest of patient’ as exemplified by the views of this researcher: ‘*Well in that case, it is like taking consent for surgery from patients. The consent is designed in such a way that any other necessary procedure apart from the one that is consented to is left to the decision of the surgeon if it is in the best interest of the patient. I just think this probably could be applied in this case, especially since you are keeping the sample,’* ***(Dr Stephanie).***

Three of the researchers (10%) opted for re-consenting. The main reasons they gave were a) if secondary use of samples would cause harm to the participants, and b) when performing procedures not specified at the initial consent process.

‘Yes, we need to go back. It is necessary to go back. From the onset, if we know that there is a need to go outside the test(s) that we specified initially, we need to seek the consent again,’ **Dr Stone**

‘If I am to give you an ideal answer, the answer would be for you to go back. That’s the ideal answer, but a more practical answer would be just to run the test and inform the individual later, if it’s not going to harm the person,’ **Dr Steel**

Three of the researchers (10%) opined that the participants’ consent could be presumed. Some researchers who preferred broad or blanket consent opined that presumed consent may not be acceptable to most potential research participants. However, if the community leaders approve of the research, and there is a high level of trust in the researcher and/or the research institution to ensure confidentiality and privacy, the consent of the community members may be presumed. This biomedical researcher implied presumed consent when he said, *‘I think that if the participant has consented that the sample should be kept, by that same token, the person is also agreeing that further study could be done on the sample. As long as it is kept confidential, then unauthorised access is not permitted’* ***(Dr Saint).***

Two of the researchers said that the ethics committee could play a role in consenting for further tests on donated samples to avoid the problems of re-consenting or tiered consenting. This is like the ‘*third-party oversight*’ model or delegated trustee. In a delegated trustee, donors can transfer consent to a trustee who is at arm’s length from the biobank, who then consents on behalf of donors (Master and Resnik, 2013; Joly et al., 2015).

‘And the research ethics board can be duly informed that this issue of re-using donors’ samples came up, this research for which you gave clearance, this is what our intentions are again. I think the regulatory body can step in and say that there is no problem and go ahead. Rather than getting back to the people,’ **Dr Mills.**

One of the researchers suggested a *dynamic consent model* that uses a social media-related application on a mobile phone. However, he felt it might not be appropriate or effective in a developing country like Nigeria due to incessant electricity disruptions, financial and economic implications, and challenges of ensuring privacy.

‘There is something we call bulk SMS now. If you have your patient’s record, you can reach them via their phone numbers. You can reach the ones that you think are eligible for the new research. You can send out bulk SMS or call them to obtain further consent.’ **Dr Misty**

### Opinions of the community leaders and community members

There was discordance in opinions between the community elders and community members. The majority (16/20; 80%) of the adult community members agreed to *blanket or broad consent,* as most of the researchers, as exemplified by this respondent, ‘*He (researcher) must have a reason for asking for the specimens, no need for re-consenting. The blood is with him. He is looking for something, he is free to conduct further tests’* ***(Flora - adult),*** but the community elders said that the community members would not agree to blanket consenting but would prefer re-consenting or multi-layered consent. The youths preferred specific consent with the re-consenting model. The youth participants’ preferences implied that the researcher obtained specific consent from them for the initial research and returned to obtain consent for another research to be performed on their biospecimens. Details of the discordant views are stated in Table 3.

**TABLE 3:** Discordant responses among study participants.

Ethical issues	Potential research participants		Community elders	Biomedical researchers
	*Adults*	*Youths*		
Type of consent process preferred	The majority preferred blanket consent	Preferred re-consenting model	The community would prefer re-consenting, except the community ruler intervened for blanket consent	*Blanket* – 12 *Broad* – 9 *Re-consenting* – 3 *Presumed* – 3 *Third-party oversight* – 2 *Dynamic* – 1 Most preferred either blanket or broad consent. The minority opted for re-consenting
Community approval versus community consent	Community approval of research is NOT consent – minority view	Community approval of research is NOT consent – majority view	Community approval is NOT consent – majority. Deviant case 1 out of 4 – the community would consent if the community ruler approved the conduct of research	Community approval of research is NOT consent – majority view Deviant cases 6 out of 30- opined that community elders’ approval constitutes consent, as the community members would agree to participate because of the respect they have for the constituted authority

The community leaders identified barriers to the blanket consent model, including a) fear of unethical use of bio-specimens (like using them for rituals), b) mistrust, and c) not thinking that the research is beneficial. The community leaders, however, opined that community leadership’s intervention might facilitate the acceptance of the broad and blanket consent models. The community leaders would have to reassure the people of the researcher’s integrity and competence to gain their trust and cooperation to achieve this.

**Elder James:** ‘It is difficult to get them to agree to storage and re-use, but if the king approves of the research, then they are assured that they are in good hands, that is when they would agree to their blood being used for other research.’

### Community approval versus community consent

The concept of what constitutes community consent, which is consent by a population group (that is, the community) or its representatives as proxy (in this case, community leaders), and its place in the conduct of research in sub-Saharan African countries has remained contentious among bioethicists (Peeters Grietens et al., 2014). Some have advanced the notion that when research is conducted in communities, and the results may ‘do harm to’ communities socially, economically, or medically, then informed and voluntary consent should be obtained from individuals and communities (Kaufman and Ramarao, 2005). Biobanking research involves the community because of the potential risks of group discrimination. It therefore calls for a stronger community-researcher rather than participant-researcher partnership. As a result, I explored the study participants’ views on the interplay between community and individual consent regarding biobanking research.

The perception among most of the researchers (80%), three of the community elders (75%) and some of the focus group participants (60%) was that individuals were free to decide whether to participate or not, irrespective of the community ruler’s approval. One of the elders said, ‘*the Oba (community ruler) does not give consent for the people. The people must still give their consent’* ***(Elder Johnson),*** and an adult male opined ‘*(That is) Correct, the Oba (community ruler) does not give consent for the people, the people must give their individual consent, the people still have the power of choice’* ***(Mackie).***

Although there was consensus among majority (80%) of the biomedical researchers that individual members of the community must give individual informed consent despite the community approval, there were discordant views from a few (six out of the 30 researchers; 20%) who said that once the community elders approved the conduct of a research, the community members would agree to participate because of the respect they have for the constituted authority but that does not preclude obtaining informed consent which, according to them, would be a formality. This latter group of biomedical researchers felt that prospective research participants would not bother considering the possible risks and benefits once the community leaders had approved the research because of the trust and confidence in the ‘gatekeeping’ ability of the community leaders. In effect, this group of biomedical researchers believed that prospective research participants would agree to donate their biospecimens because they respected the community leaders and trusted the leaders to vet the research and confirm its safety before approving it.

In addition, one of the community leaders stressed that the community members perceived the community ruler’s approval as ‘community consent’. As it is the traditional practice and cultural norm, the community is expected to comply with directives from the community ruler rather than do otherwise, irrespective of their personal opinions or beliefs. But the responses among the youth participants were contrary to this. They did not base their decisions to participate in biobanking research on communal values or community approval; rather, they demonstrated a generational shift and expressed relative solidarity, a concept already discussed in a previous publication (Ogunrin et al., 2018b).

## Discussion

This qualitative study demonstrated discrepancies in views and opinions among biobank research stakeholders regarding consent preferences for biospecimen donation. Although adult community members and most biomedical researchers preferred blanket or broad consent, the youths prefer re-consenting. The community leaders perceived that community members would not accept blanket or broad consent but would prefer re-consenting or multi-layered/tiered consent. They, however, expressed the view that approval of research by the community leadership would facilitate acceptance of blanket consent among the community members. The finding of blanket or broad consent preference among the adult community members is similar to that of an earlier Nigerian qualitative study on knowledge and attitudes to biobanking among lay persons, which showed that most respondents agreed to broad consent (Igbe and Adebamowo, 2012). This study neither categorised study participants into adults and youths, nor included clinician-researchers.

Similarly, studies from other sub-Saharan African countries that investigated the consent preference among stakeholders reported acceptance of broad consent. Tindana et al. (2020) reported that key stakeholders in Ghana, comprising genomic research implementers, ethics review board members, and community research participants, were willing to give broad consent for their samples and associated data to be used for future research purposes (Tindana et al., 2020). A cross-sectional study of 500 healthcare users in Uganda reported an overall 86.2% acceptability of broad consent for storage of their biospecimens and associated data for research purposes (Nansumba et al., 2022). It is unclear if the research participants in these studies were informed of the different consent models and allowed to choose the ones they preferred. Also, the study participants were not categorised into adults and youths to explore the discordance of views and opinions.

Discrepancies in the preferences of research participants regarding the type of consent for biobanking, as demonstrated in this study, have been reported in systematic reviews (D’Abramo et al., 2015b). A systematic review of 10 studies (comprising five from USA, one from Italy, one from Spain, one from Sweden, one from Australia, and one from Japan) showed that a Spanish study of 279 research participants reported majority (59.8%) preferred some type of limitation to consent, with 29.7% preferring to be re-contacted in case of any research on their donated bio-specimens (re-consenting) (Valle-Mansilla et al., 2010). An Australian study revealed that 85.9% of research participants accepted surrogate decision-making by regional RECs regarding the use of blood donated to the biobank (third-party oversight) (D’Abramo et al., 2015b). A study that explored the preference among US participants through 15 focus groups reported 48% opting for blanket consent at the beginning of the study, while 42% favoured being asked at the initiation of each research project. This latter study, however, observed that white people (51%) were significantly more likely than African Americans (38%) or Hispanics (40%) to favour blanket consent. Conversely, 81% of African Americans and Hispanics and 73% of white people indicated that they would have more trust in a study if they were asked to provide consent for each research project and concluded that participants wanted ongoing choices and control over access to their samples and information (Murphy et al., 2009). With respect to the sub-Saharan scenario, a recent systematic review of bioethical issues relating to informed consent in genetic and genomic research that considered consent models reported controversies and ongoing conversations over the selection of an appropriate model (broad, tiered, and dynamic) against the background of autonomy and practicality (Li et al., 2025).

In support of contrasting preferences of consent models, another US study showed that more individuals preferred broad consent (52%) over study-by-study consent models (48%), but higher preferences for study-by-study consent were observed among black non-Hispanic people and respondents with lower income and education. The discrepancies were explained by differences in the prevalence of one or more beliefs about the study. Respondents with fears about research and those who would feel respected if asked for permission opted for the study-by-study model (Platt et al., 2014). In a Jordanian study of 3,196 individuals, the majority (75.2%) preferred general consent as opposed to 16.9% that opted for the disease-specific model (Ahram et al., 2012). An Italian study showed that the public preferred broad consent (57%), with partially restricted (16%), specific (15%) and multi-layered consent (12%) being the other models of consent chosen. On the other hand, the ethics committees opted for specific consent (52%), with 8% refusing the broad consent model (Porteri et al., 2014).

A Canadian study that explored the perspectives of scientists on consent in the context of biobanking research found that the majority reported their preference for general or blanket consent, like the findings of this study. Although the Canadian scientists did not believe there was a consensus on which consent type was best, they expressed concerns that donors could need some form of assurance that nothing unethical would be done with their samples and information (Master et al., 2014). The concerns of unethical practices expressed by the scientists in the Canadian study are identical to the reasons offered by the community leaders in this study on why community members might not prefer blanket or broad consent.

In the endeavour of biobanking research, there is controversy concerning what type of consent model meets high ethical standards (Master and Resnik, 2013; Staunton and Moodley, 2013). Many biobanks have adopted the broad consent model as it is deemed a pragmatic model, but some ethicists have argued that it is not an acceptable solution to the challenges of informed consent in biobanking research. Calls for change were based on avoidance of paternalism, intentions to fulfil autonomy/liberal individualism, a desire to increase user participation, and ideas advocating the reduction of top-down governance. Hence, dynamic consenting, a model that uses modern communication strategies to inform, involve, offer choices and obtain consent to every research project, has been advocated (Steinsbekk et al., 2013b). One of the biomedical researchers in this study opted for the dynamic model. This model is, however, costly, technology-based, and requires infrastructural support; these are conditions that may constitute challenges in low-resource countries (Afolabi et al., 2014).

An important issue when considering consent preferences for biobanking research in developing African countries is the concept of ‘community consent’, which is a scenario where community elders’ approval for the conduct of research is accepted as consent for individual participants because of respect for traditional authority; a situation where individuals are not allowed to express their autonomous choices. This concept does not conform to informed consent as it is known in Western research scenarios. Sometimes, the decision makers in this situation may not be community leaders but religious leaders and African traditional priests (for example, in Yoruba culture, they are referred to as *‘babalawo’*). They are considered intermediaries between the living and the heavenly deities. The Christian religious leader or the Imam of the Islamic faith is an intermediary between the people and God, while the traditional priests serve as intermediaries between the people and the ancestors or deities such as Ogun, Sango, Orunmila, Obatala, and so on (Ogungbile, 1997). In African communities, there is a belief that Western medicine or research can provide neither a cure nor an explanation for certain diseases among Africans because causation is explained in terms of misfortune in the relationship between the individual and the social, natural, and spiritual environments (Chukwuneke et al., 2012). Therefore, people suffering from a disease whose origin has been attributed to supernatural causes, and their families, may seek explanation and possible cure for the disease at fetish shrines, diviners or spiritualists. Thus, it is not unusual to encounter communities in Africa where, before a decision is taken concerning research participation or treatment options, the opinion of a religious leader or spiritualist is sought after, serving as a ‘third-party’ decision maker (Awusabo-Asare and Anarfi, 1997).

Controversies surround the concepts of ‘community consent’ and ‘third-party consent’ because these practices do not conform to the fundamental principles of informed consent, namely, autonomy and the individual’s right to make a rational, uncoerced choice. In practice, however, the responsibility of research decision-making may rest with a third party even if the participant is competent in most African societies. It is important to recognise the ethical and cultural problems associated with this scenario to avoid conflicts and tensions and appreciate the benefits so that it can be properly applied to encourage research participation while minimising risks. It is also important to note that Western bioethical concepts may be difficult to implement in sub-Saharan African cultural settings due to ethical pluralism. Shaibu (2007) narrated his experience of conducting research in Botswana and noted the tensions and conflicts that arise from adhering to the Western conceptualisation of bioethics. He stressed the need to be culturally sensitive when conducting research among indigenous populations. Cultural practices required the need to exercise discretionary judgment guided by respect for the culture and decision-making protocols of the research participants (Shaibu, 2007). This observation was corroborated by a qualitative Ghanian study that pilot-tested an informed consent framework in four communities and concluded that, rather than reject standard consent models, a context-sensitive adaptation that enables ethical and legitimate recruitment could be adopted (Appiah et al., 2026). It is becoming common practice in some developing African communities that have embraced responsive communitarianism, as opposed to authoritarian communitarianism, to emphasise individual consent when participating in research, including biobanking (Etzioni, 2011; Jegede, 2009). I opine that community consent is an important concept for effective community engagement, but it should not replace individual consent; instead, they should complement each other. This stance is supported by a recent scoping review on the ethico-cultural and implementation challenges associated with the individual-based informed consent model in the African context. This review concluded that ‘a pluriversal, context-specific, multi-step informed consent model that critically harmonises the cultural values of the local population can minimise ethics dumping, safeguard research integrity, and promote respectful engagement’ (Appiah et al., 2024).

**FIGURE 1:**
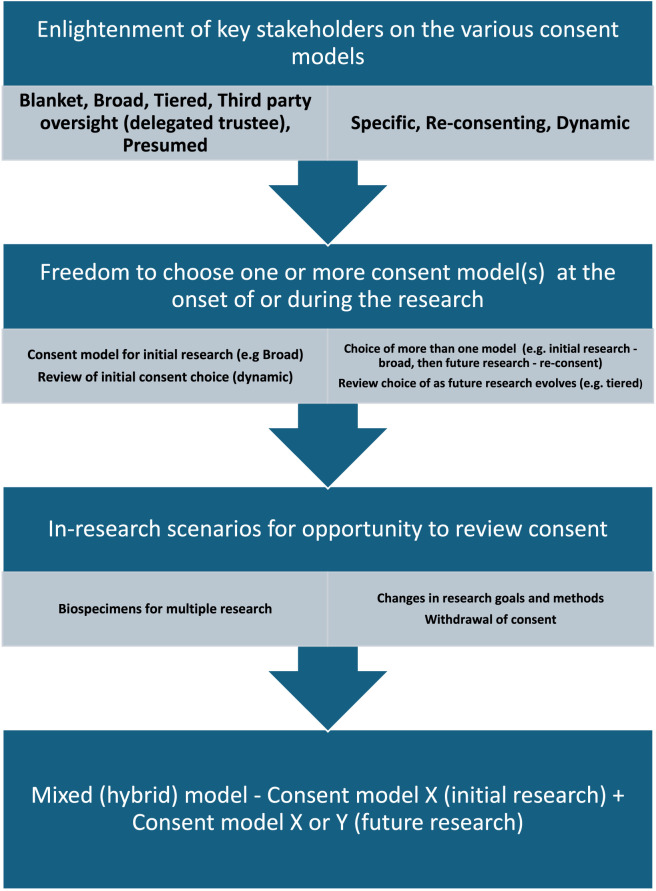
Components of Mixed or Hybrid model. Choice of more than one model (e.g. initial research - broad, then future research - re-consent). Review consent choice as future research evolves (e.g. tiered)

**FIGURE 2:**
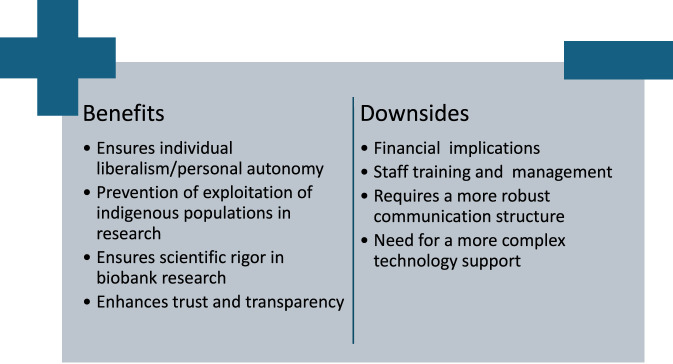
The pros and cons of a mixed model.

To prevent ethical conflicts that may arise from the diverse consent preferences among biobank research stakeholders, I propose a mixed or hybrid consent model. A mixed model will facilitate the combination of the characteristics of alternative consent models and allow prospective research participants or biospecimen donors to express their preferences to the biobank. The freedom to choose a consent model allows individuals to express their autonomy, personal beliefs, and cultural practices. Hence, biobank researchers are responsible for informing donors about the details of research, obtaining their consent preferences, and designing feasible consent processes that align with the choices of the research participants. This model first involves informing stakeholders, including researcher-clinicians and research participants, of the various consent models, their benefits and drawbacks, and including them as options for participants to consider during the informed consent process. Second, it allows research participants or biobank donors to choose, either at the beginning of or during biobank research, the consent models they prefer for the initial and future research on their biospecimens. The implementation of the mixed or hybrid consent model, especially if it includes re-consenting or dynamic consent, facilitates active participation of biobank donors in the consent process, respects their autonomy, and provides the opportunity for feedback on their results. Figure 1 illustrates the stages and components of the mixed or hybrid model, and Figure 2 shows the benefits and downsides of the model.

### Study limitations

This is a single-site study at a Yoruba-speaking setting; therefore, future research involving multiple sites and communities with diverse socio-cultural characteristics is desirable. Although all biomedical researchers who participated in this study were familiar with genomic and biobank research, it was limited by the underrepresentation of researchers actively involved in biobank research. Hence, future studies that explore the views of biomedical researchers actively involved in biobank research will provide a better understanding of their consent alignments.

## Conclusions and recommendations

The findings of this study showed that participants within the same community may prefer different consent models and demonstrated discordant views on consent preferences among researchers, community gatekeepers and community members. Thus, researchers would need to exercise flexibility in the adoption of consent models for their biobank projects. It may be preferable to offer participants the opportunity to make their choices after ensuring that they understand the research details. A mixed or hybrid consent process would provide prospective biobank research participants with options. The role of a community member on ethics committees is important to the implementation of biobanking research, as they advise on cultural norms and practices that may affect consent preferences. Although it is already a standard practice to appoint community representatives and religious leaders as members of ethics committees, their roles become more relevant when reviewing biobanking research protocols. I recommend that representatives of community professional or social groups be included as members of the community advisory board to facilitate understanding of communal norms and practices, enhance dissemination of research information, recruitment of research participants, and foster trust and bonding with community members. To harmonise the governance of emerging biobanks in sub-Saharan African countries, I recommend a robust, culturally sensitive, and context-specific, mixed or hybrid consent model that reflects the choices of prospective research participants, which may be complemented by community consent, and incorporates the preferences of other biobank research stakeholders.

## Data Availability

The raw data supporting the conclusions of this article will be made available by the authors, without undue reservation.
