# Supplementary material for: Biobank consent preferences and implications for a mixed consent model: biomedical researchers vs. community stakeholders in a semi-urban Yoruba community, Nigeria
Source: Front Genet. 2026 Jun 3;17:1779086. doi: 10.3389/fgene.2026.1779086 (PMC13271731; doi:10.3389/fgene.2026.1779086)
Supplement: Supplementary file 1 [file Supplementaryfile1.docx]

**APPENDICES**

**A: Interview topic guide**

**Demographic data of the study participants**

This shall be obtained using a prepared format as presented below:

Age ………………. (years) Sex ………………. (Male/Female)

Domicile …………………………………………………………………………………………………………………

Level of education ……………………………. (No education, Home tutor, Primary, Secondary, Tertiary)

Ethnicity ………………………………… (Yoruba, Igbo, Hausa, others)

Religion …………………………………. (Islam, Christianity, African Traditional, others)

Level of Income …………………… (average per year in Naira)

Area of research …………………………… (for biomedical researchers)

Role in the community ……………………………… (for opinion leaders or community elders)

| S/N | Topic of interest | Question |
| --- | --- | --- |
| 1. | **Process of decision making in the community** | How does an individual make decision to participate in a venture/project/undertaking within the community? If the venture is a research, how will he/she decide to participate? Has there been any change in the decision making process? If yes, why and when? Is there any input to that decision from the community authority/family members/others? If yes, what type of input? Are there other factors that influence such decision? Which are these factors, and how they do influence your decision making ability? |
| 2. | **Knowledge of genomic research** | Have you ever heard of genomic research?  If yes, could you tell me what genomic research looks at?  If no, (this is what it is - genomic research looks at the human genome which is the complete makeup of the human DNA that predict chances of developing diseases in life or pattern of inheritance of diseases). |
| 3. | **Impact of cultural and religious beliefs on research participation** | Do you think that religion can affect participation of individuals in research? How does it affect participation?  Does your culture encourage participation in research? Are there cultural practices or norms that affect participation of community members in research? What are these practices? How do they affect research participation? Are there norms that allow or disallow different genders or age groups to participate in research? |
| 4. | **Knowledge of informed consent and who gives the consent** | Could you tell me what ‘informed consent’ (agreeing to participate in research or any other project after you have received full information on what the project is about) means? How much information will you need to agree to participate in genomic research? Can you give me some examples of such information? What do you think the process should entail? In genomic research, do you think informed consent is important? In genomic research, specimens (e.g. blood samples) may be used for other purposes apart those initially given. This will be explained in the study information given to potential study participants. Do you think your consent for the initial purposes is sufficient to cover for the new purposes? If not, why? What will you want the researcher to do? |
| 5. | **Awareness of benefits and risks of research** | Do you think research is associated with any risk? What of genomic research? What are these risks? Can you give examples of such risk associated with research within your community (if there is any)? Are there benefits associated with research? What of genomic research? Can you give examples of such benefits associated with research (if there is any)? How did you know about these risks and benefits? |
| 6. | **Appreciation of importance of ethics in research in the community** | Ethics regulate research by reviewing research protocol, monitoring implementation and ensuring researchers do what is right. Do you think this is necessary? Can you tell me the reasons for your answer? |
| 7. | **Perception of export of donated specimens, ownership of such specimens and desirability for feedback following analysis of specimen** | If you were asked, would you provide consent for your specimen collected for research purposes be taken somewhere else in Nigeria for analysis? If not, why? Would you provide consent for your specimen to be analyzed outside Nigeria? If not, why?  Who do you think own specimens collected for research? Why do you think so? Would you want to receive the results of the analysis on your specimen? How would you want to receive the results? Do you think your family members should be informed of the result if it may impact on their health? Why do you think so? |
| 8. | **Perception of community participation in research**  ***(I will explore how much trust the community has in researchers)*** | What does the community expect from researchers who want to conduct research in the community? Are there rules or customary norms they need to comply with?  Will the community want to be part of the conduct of a research? If not, why? If yes, which part of the research will the community want to have an input? How will you want researchers to go about involving the community in their research? Can you give me examples? |
| 9. | **Awareness and adequacy of the national code of health research ethics in conduct of genetic and genomic research (for biomedical researchers)** | Are you aware of the National Code of Health Research Ethics? How did you know about it?  Is there any guideline for conduct of genomic research in Nigeria? Do you think the Code, as it is presently, sufficiently covers the conduct of genomic research in Nigeria? Is there need for any guideline for genomic research? What are the ethical issues you will like a guideline on genomic research in Nigeria to address? |

Thank you for choosing to participate and for your time.

**Appendix vi**

**B: Discussion topic guide**

**Demographic data of the study participants to include gender, level of income, ethnic orientation, religious affiliation, age and level of education**

This shall be obtained using a prepared format as presented below:

Age ………………. (years) Sex ………………. (Male/Female)

Domicile ……………………………………………………………………………

Level of education ……………………………. (No education, Home tutor, Primary, Secondary, Tertiary)

Ethnicity ………………………………… (Yoruba, Igbo, Hausa, others)

Religion …………………………………. (Islam, Christianity, African Traditional, others)

Level of Income …………………… (average per year in Naira, if applicable)

Role in the family ……………………………… FGD Category ……………………………

| S/N | Topic of interest | Question |
| --- | --- | --- |
| 1. | **Knowledge of genomic research** | Have you heard of ‘research’ before? Can you tell me what you understand by the word ‘research’? Have you heard of genomic research before? What does genomic research mean? How did you know about it? |
| 2. | **Importance of ethics in research in their communities** | Do you know what ethics mean? Do you think ethics is important in research? What is the significance of ethics in research? Can you explain why it is important? |
| 3. | **Process of decision making in the community** | If you have to participate in research how will you make a decision? Has there been any change in the decision making process? If yes, why and when? Is there any input to that decision from the community authority/family members/others? Are there other factors that influence your decision? |
| 4. | **Awareness of benefits and risks of research** | Do you think research is risky? What of genomic research? Can you tell me some of these risks? Can you give examples (if there is any)? Do you think there are benefits associated with research? What of genomic research? Can you give examples (if there is any)? How did you know about these benefits and risks? |
| 5. | **Impact of cultural and religious beliefs on research participation** | Does your religion affect participation of individuals in research? How does it affect participation? Does your culture encourage participation in research? Are there cultural practices or norms that affect your participation in research? What are these practices? How do they affect your participation? Do you think your gender/age group put you at a disadvantage as regards research participation? |
| 6. | **Knowledge of informed consent and who gives the consent** | What does ‘informed consent’ (agreeing to participate in research or any other project after you have received full information on what the project is about) mean to you? How much information will you need to give your consent for participation in genomic research? What do you think the process should entail? In genomic research, do you think informed consent is important? In genomic research, specimens may be used for other purposes apart those initially given. Do you think your consent for the initial purposes is sufficient to cover for the new purposes? If not, why? What will you want the researcher to do? |
| 7. | **Perception of export of donated specimens, ownership of such specimens and desirability for feedback following analysis of specimen** | Will you allow your specimen collected for research purposes be taken somewhere else in Nigeria for analysis? Will you export outside Nigeria for analysis? If not, why? Who do you think own specimens collected for research? Why do you think so? Will you want results of the analysis on your specimen report back to you? How will you want to receive the results? Do you think family members should be informed of the result if it may impact on their health? Why do you think so? |
| 8. | **Perception of community participation in research** | What do you expect from researchers who want to conduct research in your community? Do you know of any customary norms they need to comply with? Will you want to have a say in which type of research is conducted in your community? Which part of the research will you want to have a say? How will you want researchers to go about involving you in their research? |

Thank you for choosing to participate and for your time.
